# Quenching-Chemiluminescence Determination of Trace Amounts of **l**-Tyrosine Contained in Dietary Supplement by Chemiluminescence Reaction of an Iron-Phthalocyanine Complex

**DOI:** 10.1155/2012/520248

**Published:** 2012-03-27

**Authors:** Takao Ohtomo, Shukuro Igarashi, Yoshitaka Takagai, Osamu Ohno

**Affiliations:** ^1^Department of Biomolecular Functional Engineering, Faculty of Engineering, Ibaraki University, 4-12-1 Nakanarusawa, Hitachi, Ibaraki 316-8511, Japan; ^2^Faculty of Symbiotic Systems Science, Cluster of Science and Technology, Fukushima University, Kanayagawa 1, Fukushima 960-1296, Japan

## Abstract

The chemiluminescence (CL) signal immediately appeared when a hydrogen peroxide solution was injected into an iron-phthalocyanine tetrasulfonic acid (Fe-PTS) aqueous solution. Moreover, the CL intensity of Fe-PTS decreased by adding l-tyrosine. Based on these results, the determination of trace amounts of l-tyrosine was developed using the quenching-chemiluminescence. The calibration curve of l-tyrosine was obtained in the concentration range of 2.0 × 10^−7^ M to 2.0 × 10^−5^ M. Moreover, the relative standard deviation (RSD) was 1.63 % (*n* = 5) for 2.0 × 10^−6^ M l-tyrosine, and its detection limits (3*σ*) were 1.81 × 10^−7^ M. The spike and recovery experiments for l-tyrosine were performed using a soft drink. Furthermore, the determination of l-tyrosine was applied to supplements containing various kinds of amino acids. Each satisfactory relative recovery was obtained at 98 to 102%.

## 1. Introduction

In vivo l-tyrosine is known as a substance with a reductive action. The l-tyrosine is a substance that generates a neurotransmitter such as adrenaline, norepinephrine (noradrenaline), or dopamine and becomes the thyroid hormone that controls the metabolism and is a precursor of melanin [1, 2]. Therefore, the development of a method to determine small amounts of l-tyrosine is important in the medical and pharmacology fields. Moreover, the intake of l-tyrosine is also necessary for the preservation of health and is contained in nutrition supplements. Recently, the guarantee of its quality and analysis has become especially important problems in the nutritional science field.

l-tyrosine has been determined by various methods. For example, ultraviolet absorptiometry at the wavelength of 280 nm [3] and the Lowry method using the phenol reagent are known as a general determination analysis methods [4], but both have too low a sensitivity. Recently, many methods have been reported. They are as follows: spectrophotometry using the phenylalanine ammonia-lyase enzyme reaction [5], fluorimetry using the 1,5-bis(4,6-dichloro-1,3,5-triazinyl-amino) synthesis reaction [2] or using the Mo(VI)-phenyl-fluorone quenching reaction [6], chemiluminescence (CL) using the oxidation reaction of K_3_Fe(CN)_6_ and KMnO_4_ [7–9], cyclic voltammetry using the multiwalled carbon nanotube/4-amino-benzenesulfonic acid film-coated glassy carbon electrode oxidation reaction [10], UV detection of microcolumn electrophoresis [11], and HPLC-FL [12]. However, only a few rapid, simple, and highly sensitive analyses of l-tyrosine are known.

The CL method is highly sensitive, and the setup is simple and has the advantage that the measurement time is short. The CL analysis of various samples is performed in many fields such as biochemistry, clinical chemistry, and environmental chemistry for this reason. However, the absolute number of high sensitive and high functional CL substance is a few although the CL system using luminol or peroxyoxalate has been many reported [13, 14]. Therefore, the appearance of a high CL substance is strongly desired. Up to now, the authors have reported about the chlorophyll CL [15] and the iron-chlorophyllin complex CL [16]. In these CLs, each compound itself has multifunctions such as a luminescence substrate, a catalyst, and an energy donor. Moreover, in the iron-chlorophyllin complex CL, the luminescence was quenched when L-ascorbic acid was made to coexist in an oxidation reaction with hydrogen peroxide. The quenching-CL determination of 10^−8^ M for L-ascorbic acid was developed [17]. On the other hand, the CL phenomenon with respect to the iron-phthalocyanine tetrasulfonic acid (Fe-PTS) was reported [18, 19]. The Fe-PTS complex had multi-CL functions similar to the iron-chlorophyllin complex, because the Fe-PTS as a luminescence substrate is the analog of an iron-chlorophyllin complex. In this paper, as a result of examining the Fe-PTS CL system, the coexistence of l-tyrosine significantly influenced the CL signal intensity. The quenching-CL method of l-tyrosine with Fe-PTS was then developed, especially, the determination of tyrosine in soft drink and dietary supplement were examined. The details are described as follows.

## 2. Experimental

### 2.1. Reagents

Iron-phthalocyanine tetrasulfonic acid was obtained from Aldrich (Milwaukee, WI, USA). Sodium tetraborate (Borax) was obtained from the Kanto Chemical Co. (Tokyo, Japan). Hydrogen peroxide was obtained from Wako Pure Chemicals Industries. (Osaka, Japan). The l-tyrosine was obtained from the Tokyo Chemical Industry Co. (Tokyo, Japan). The practical samples were obtained from a soft drink (Amino supli, Kirin beverage Co., Tokyo, Japan) and a dietary supplement (Amino body, Orihiro, Gunma, Japan). All the other reagents were of analytical grade.

### 2.2. Instrument

A GENELIGHT200S (the spectra range of PMT: 400–570 nm; Microtec NITI-ON, Chiba, Japan) was used for the CL measurements. The pH was obtained using an F-51 (HORIBA, Kyoto, Japan). The distilled water was obtained using a SWAC-500 (Shimadzu Co., Kyoto, Japan).

### 2.3. Experimental Procedure

#### 2.3.1. Standard Procedure Concerning the Determination of l-Tyrosine

A 300 *μ*L sample of water containing l-tyrosine was injected into a glass cell using a microsyringe. A 100 *μ*L pH buffer solution and 50 *μ*L iron-phthalocyanine complex solution were added and mixed. Next, the cell was then placed into the cell holder of the CL detection equipment. A 50 *μ*L aliquot of hydrogen peroxide was then injected using a microsyringe and the CL signal was measured. The determination was based on the following equation. The ΔCL = CL_0_−CL*_s_*, where ΔCL is the difference in the CL signal intensity, CL_0_ is the intensity of the l-tyrosine additive-free sample, and CL*_s_* is the intensity of a certain amount of l-tyrosine contained in the sample. 

#### 2.3.2. Spike and Recovery Experiments of l-Tyrosine in Soft Drink

A 1.0 mL soft drink was diluted to the marked line with distilled water in a 50 mL volumetric flask. Next, 5 mL of a 6.7 × 10^−6^ M l-tyrosine solution was added to 5 mL of this soft drink. The measurement was done using the standard procedure concerning the determination of l-tyrosine.

#### 2.3.3. The Determination of l-Tyrosine in Dietary Supplement

A 0.25 g sample of the tablet (the content of l-tyrosine is 6.00 mg) was dissolved in 15 mL of a 1.0 M sodium hydroxide solution and then was diluted to the marked line with distilled water in a 250 mL volumetric flask. A 1.4 mL of this solution was diluted to the marked line with distilled water in a 10 mL of volumetric flask, and the CL signal was measured using the standard procedure concerning the determination of l-tyrosine.

## 3. Results and Discussion

### 3.1. Consideration of CL Reaction

 It was examined whether l-tyrosine works as H_2_O_2_ scavenger. Since l-tyrosine is a reducing agent, naturally, authors think that it may work as H_2_O_2_ scavenger. In a previous paper [17], it is because the similar quenching-CL phenomenon by L-ascorbic acid was observed in iron-chlorophyllin/H_2_O_2_/L-ascorbic acid.

**Figure 1:**
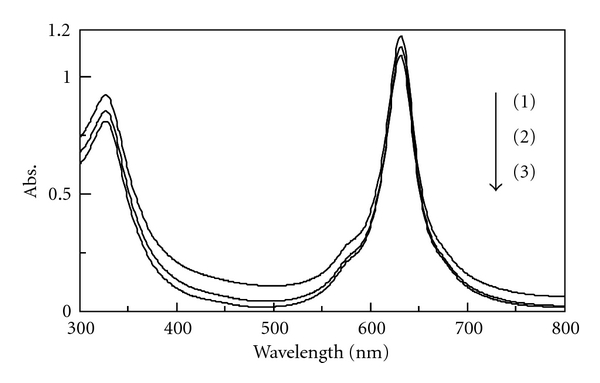
Absorption spectral change of Fe-PTS. [Fe-PTS]*_T_*  = 4.0 × 10^−5^ M, pH = 10, Signal: (1) [l-tyrosine]*_T_*  = 0 M, (2) [l-tyrosine]*_T_*  = 1.0 × 10^−6^ M, (3) [l-tyrosine]_T_ = 5.0 × 10^−5^ M.

It was examined whether l-tyrosine works as a ligand of Fe-PTS. The absorption spectral change of Fe-PTS is shown in Figure 1. The absorbance of Fe-PTS was decreased with increasing l-tyrosine concentration. From this result, it is thought that l-tyrosine works as a ligand of Fe-PTS.

 It was examined whether l-tyrosine might work as a quencher (energy transfer type). Authors performed Stern-Volmer plot. Stern-Volmer plot from 2.0 × 10^−7^ to 2.0 × 10^−6^ M in the range of concentrations of l-tyrosine became linear. Therefore, Stern-Volmer plot showed that l-tyrosine worked as a quencher.

 In conclusion, the function of l-tyrosine in the CL phenomenon is presumed form the above to work compositely and to happen.

### 3.2. Procedure Optimization

Based on the difference between the CL intensity of the blank reaction and the CL intensity for the 2.0 × 10^−6^ M l-tyrosine concentration that was the highest, each condition was optimized.

#### 3.2.1. Influence of Fe-PTS Concentration

The concentration of the Fe-PTS was varied in the concentration range of 1.0 × 10^−5^ M to 5.0 × 10^−5^ M. The CL intensity increased with the increasing concentration of Fe-PTS. The optimal concentration of Fe-PTS was 4.0 × 10^−5^ M according to the procedure optimization. 

#### 3.2.2. Influence of Hydrogen Peroxide Concentration

The concentration of the hydrogen peroxide was varied in the range of 1.6 × 10^−3^ M to 8.0 × 10^−3^ M. As a result, the maximum CL_0_ intensity could be measured at 6.4 × 10^−3^ M hydrogen peroxide. However, the maximum ΔCL could be obtained at 3.2 × 10^−3^ M hydrogen peroxide according to the procedure optimization.

#### 3.2.3. Influence of pH

**Figure 2:**
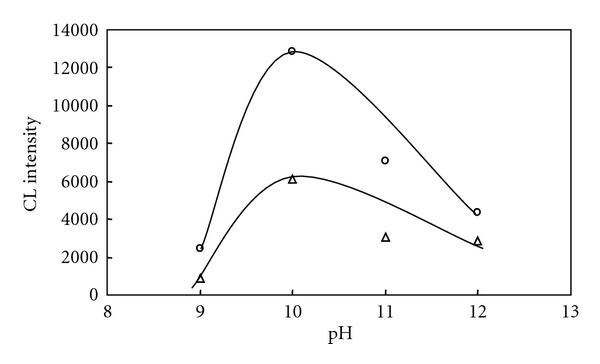
Influence of pH (PMT voltage: 730 V). [Fe-PTS]*_T_*  = 4.0 × 10^−5^ M, [H_2_O_2_]*_T_*  = 3.2 × 10^−3^ M. ∘: [l-tyrosine]*_T_*  = 0 M, Δ: [l-tyrosine]*_T_*  =2.0 × 10^−6^ M.

The relationship of the CL intensity and pH is shown in Figure 2. The pH was varied within the range of 9 to 12. The maximum CL intensity according to the pH change was observed at pH 10. The optimal pH is 10 according to the procedure optimization.

### 3.3. Calibration Curve

**Figure 3:**
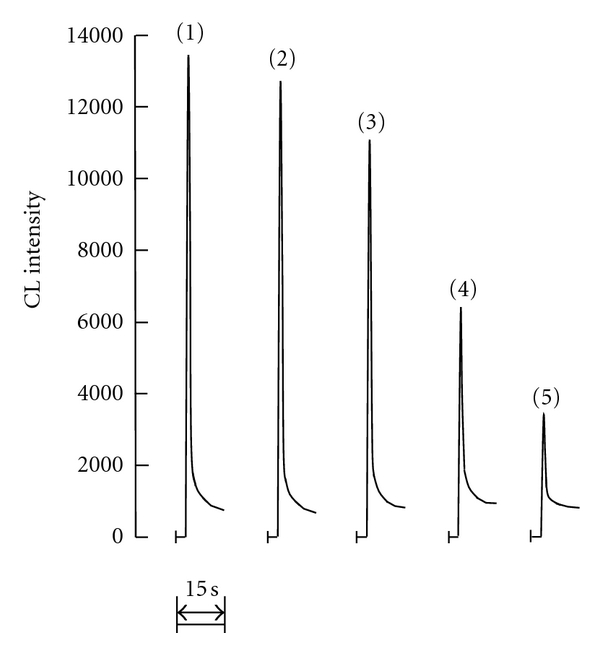
Relationship between l-tyrosine concentration and CL intensity (PMT voltage: 730 V). [Fe-PTS]*_T_*  = 4.0 × 10^−5^ M, [H_2_O_2_]*_T_*  = 3.2 × 10^−3^ M, pH = 10. Curves in various concentration (M) of l-tyrosine: (1) 0, (2) 2.0 × 10^−7^, (3) 5.0 × 10^−7^, (4) 2.0 × 10^−6^, and (5) 5.0 × 10^−6^.

The l-tyrosine was measured using the CL of Fe-PTS. The CL intensity quantitatively decreased when the concentration of the coexisting l-tyrosine increased. The CL signal for various l-tyrosine concentrations is shown in Figure 3.

In the calibration curve of l-tyrosine, the relationship obtained between the concentration range of 2.0 × 10^−7^ M to 2.0 × 10^−5^ M of l-tyrosine, and the difference in the CL intensity (ΔCL) was *y* = 2544 ln(*x*) + 39864, where *y* is ΔCL and *x* is the l-tyrosine concentration [M]. The correlation coefficient was 0.981, the RSD was 1.63% (*n* = 5) at a 2.0 × 10^−6^ M l-tyrosine concentration, and the detection limit (3*σ*) was 1.81 × 10^−7^ M. Moreover, the result of determination of tyrosine of enantiomer is shown in Table 1.

### 3.4. Influence of Foreign Substances

The interference by foreign substances was examined. This result is shown in Table 2. As for the allowable limit, the change in time was within ±5% based on the difference in the CL intensity with the blank when no foreign substances were added. This was compared to the molar ratio with l-tyrosine. As a result, L-ascorbic acid could be present up to 5-fold without affecting the results of the test. Also, although Ni^2+^ and Co^2+^ could be present up to equivalent without affecting the results of the test and Cu^2+^ could be present up to 0.5-fold without affecting the results of the test, they could be masked up to 10-fold with the addition of EDTA as a masking reagent. Also, L-cysteine could be present up to 0.5-fold without affecting the results of the test. It is reported that L-cysteine and Zn^2+^ form a complex under neutral and alkaline conditions [20]. As a result of the experiment, L-cysteine was masked up to 100-fold with the addition of Zn^2+^ as a masking reagent. The proposed method can then be comparatively said to be selective as an analytical method for l-tyrosine.

### 3.5. Comparison with Other Analysis Methods

The proposed method and other methods were compared. The proposed method (procedure time: approximately 3 min) was proved to be rapid and simple, but the detection limits (detection limits: 1.81 × 10^−7^ M) are poor for fluorophotometry by the synthesis reaction with 1,5-bis(4,6-dichloro-1,3,5-triazinyl-amino) (detection limits: 6.8 × 10^−8^ M, procedure time: 30 min) [2]. Also, the proposed method was rapid, simple, and highly sensitive compared to spectrophotometry using the phenylalanine ammonia-lyase enzyme reaction (detection limits: 5.0 × 10^−6^ M, procedure time: 2 h) [5] and quenching-fluorophotometry using Mo(VI)-phenyl-fluorone (detection limits: 5.2 × 10^−7^ M, procedure time: 5 min) [6]. 

### 3.6. The Determination of the l-Tyrosine in Dietary Supplement

The determination of l-tyrosine in dietary supplement is shown in Table 3.

The l-tyrosine in a soft drink was determined. The determined concentration of l-tyrosine was 1.96 × 10^−6^ M for 2.00 × 10^−6^ M, and the recovery was 98%. Moreover, the l-tyrosine in medicine (supplement) was determined. The indicated value of the components in the supplement was measured by the Japan Food Research Laboratories, and the various amino acids were measured by the amino acid analytical method using ninhydrin reaction. The experimental value that had been obtained by the measurement was 2.04 × 10^−6^ M for the tabulated one of 2.00 × 10^−6^ M l-tyrosine. Satisfactory results for each sample were obtained using this method.

## 4. Conclusion

In this study, rapid, simple, and highly sensitive analysis method of trace amounts of l-tyrosine based on the CL reaction of Fe-PTS was developed. In the future, a new FIA CL-detection will be developed for application to multisamples containing trace amounts of l-tyrosine.

## Figures and Tables

**Table 1:** Determination for isomer of tyrosine.

Substance	Detection limit (M)	RSD (*n* = 5)
l-tyrosine	1.81 × 10^−7^ M	1.63%
d-tyrosine	9.69 × 10^−8^ M	1.99%

**Table 2:** Influence of foreign substances.

Foreign substance	[Foreign substance]/[l-tyrosine]
Glutamine, methionine, serine, threonine, alanine, glycin, isoleucine, glucose, fructose, xylose, saccharose, raffinose, levulose, lactulose, galactose, nicotinic acid, nicotinamide, sodium pantothenate	1000
Asparagine, lysine, phenylalanine, histidine, leucine, proline	500
Ca^2+^, Zn^2+^, Mn^2+^, valine, aspartic acid cysteine*	100
Se^4+^, V^5+^, Al^3+^, arginine, pyridoxine hydrochloride, thiamine hydrochloride	50
EDTA, Ni^2+^,* Co^2+^,* Cu^2+^,* Fe^3+^, glutamic acid, riboflavin, folic acid	10
Cystine, tryptophan, ascorbic acid	5

Tolerance limit is within ±5% error for CL intensity when [l-tyrosine]*_T_*  is 2.0 × 10^−6^ M.

*[EDTA]*_T_*: 2.0 × 10^−5^ M was added as a masking reagent.

*[Zn^2+^]*_T_*: 2.0 × 10^−4^ M was added as a masking reagent.

**Table 3:** Determination of l-tyrosine of dietary supplement.

Sample	Added (M)	Found (M)	Recovery (%)	RSD (*n* = 5)
Sample A^a^ (soft drink)	2.00 × 10^−6^ M	1.96 × 10^−6^ M	98%	2.42%
Sample B^b^ (component indicated value of supplement)*	—	2.04 × 10^−6^ M (2.00 × 10^−6^ M)*	—	2.95%

^a^Component: aspartic acid, arginine, lysine, glutamic acid, glycine, proline, alanine, leucine, isoleucine, valine, ornithine, citrulline.

^b^Component: protein, lipid, carbohydrate, sodium, vitamin B_1_, vitamin B_6_, twenty kinds of the amino acid including the l-tyrosine.

*Indicated value of medicine.
